# Effect of Protease Inhibitors in Healing of the Vaginal Wall

**DOI:** 10.1038/s41598-019-48527-0

**Published:** 2019-08-26

**Authors:** Maria Florian-Rodriguez, Kathleen Chin, Jennifer Hamner, Jesus Acevedo, Patrick Keller, R. Ann Word

**Affiliations:** 1 Department of Obstetrics and Gynecology Division of Female Pelvic Medicine and Reconstructive Surgery, University of Texas Southwestern Medical Center, Dallas, Texas USA; 2 Cecil H. and Ida Green Center for Reproductive Biology Sciences, University of Texas Southwestern Medical Center, Dallas, Texas USA

**Keywords:** Urogenital reproductive disorders, Mechanisms of disease, Experimental models of disease

## Abstract

Impaired elastogenesis and increased degradation of elastic fibers has been implicated in the pathogenesis of pelvic organ prolapse. Loss of the elastogenic organizer, fibulin-5 (FBLN5), leads to pelvic organ prolapse in mice. The objective of this study was to investigate the regulation of FBLN5 after surgical injury of the vaginal wall using the rat as a preclinical animal model. Both endogenous and recombinant FBLN5 were degraded after surgical injury. Estrogen did not alter the dramatic loss of vaginal FBLN5 in the acute phase after injury (12–48 h), but resulted in rescue of the poor recovery of FBLN5 levels in the late phase (7 d) of healing in ovariectomized animals. In contrast with estrogen, the general MMP inhibitor, actinonin, abrogated injury-induced degradation of FBLN5 significantly. Further, actinonin rescued the negative effects of injury on biomechanics, histomorphology, and elastic fibers. Control of excessive matrix degradation by local application of actinonin at the time of surgery may lead to improved elastic fiber regeneration and wound healing, thereby potentially enhancing pelvic floor recovery after reconstructive surgery for prolapse.

## Introduction

Pelvic organ prolapse (POP) is a common disorder for which approximately 400,000 operations are performed per year^1,2^. This disorder can have a severe impact in quality of life, including sexual function^3^. Our lack of understanding in the pathogenesis of POP is compounded by high failure rates of reconstructive surgery for POP. Failure rates after pelvic reconstruction surgery have been described to be as high as 56%; with the highest rates reported after native tissue repairs^1,4,5^. It has been suggested that impaired recovery of pelvic organ support after vaginal delivery may lead to prolapse later in life as compensatory mechanisms fail during aging, weight gain, and menopause^1,6^. These same factors may be responsible for impaired wound healing after reconstructive surgery, thus contributing to the unacceptable high recurrence rates of POP after surgery.

Impaired elastogenesis and increased degradation of elastic fibers leads to pelvic organ prolapse in mice^7–13^. Elastic fibers provide structural integrity in the vaginal wall and paravaginal attachment sites, protecting against mechanical strain. FBLN5 is a key matricellular glycoprotein that promotes elastogenesis^14,15^ and inhibits the matrix degrading protein MMP-9^7^. In tissue-specific conditional knockout mice, it was shown that FBLN5 is important for protection and recovery of the pelvic floor after parturition^16^. In humans, FBLN5 expression is decreased and MMP-9 increased in connective tissues of the pelvic floor from women with prolapse^7,17,18^. These observations support the hypothesis that pelvic organ prolapse is in part due to a disorder of the extracellular matrix in which matrix regeneration and degradation are not balanced. Many studies have addressed the role of collagen homeostasis after surgical injury in the vagina^19^, but few have examined the impact of injury on elastogenesis or elastic fiber degradation.

FBLN5 mediates endothelial cell adhesion, regulates cell growth and motility, and it’s essential for elastic fiber formation. Lee *et al*. used a gene therapy approach to deliver FBLN5 to rabbit ear full-thickness dermal wounds and study FBLN5 role in wpund healing^20^. Interestingly, FBLN5 promoted wound healing *in vivo* with considerable net increase (∼50%) in both newly formed granulation tissue volume and wound closure. FBLN5 expression also stimulated substantial expression of collagen in dermal wounds. The results suggest that expression of the extracellular matrix protein FBLN5 enhances expression oftype I collagen *in vivo*. Previous experiments indicated that injection of recombinant FBLN5 in knockout mice rescued MMP-9 activation up to 1 month after treatment^21^. In addition to FBLN5, estrogen has been postulated as an adjunct to pelvic floor reconstructive surgery^19,22–25^. Here, we investigate the regulation of FBLN5 after surgical injury of the vaginal wall using the rat as a preclinical animal model. Further, we tested the hypothesis that estrogen or local protease inhibitors at the time of surgery may alter FBLN5 and matrix proteins as a function of time after injury.

## Results

### Effect of injury on FBLN5 content in the vaginal wall

**Figure 1:**
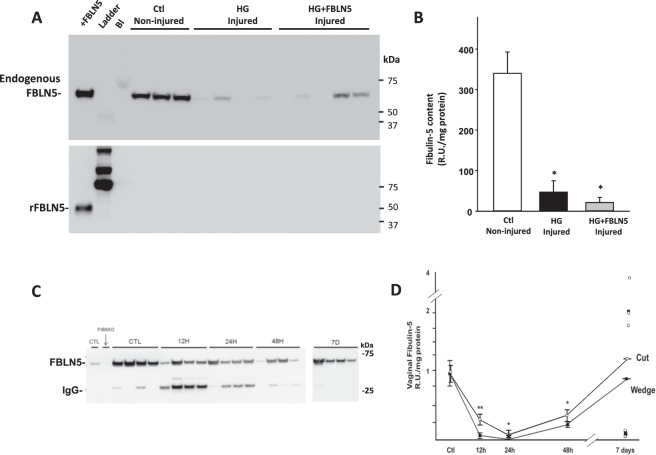
Effect of vaginal wall injury on FBLN5. Nulliparous young rats were either not injured or injured as described in Materials and Methods. Injured rats were treated with hydrogel (HG) alone or HG + FBLN5 (10 µg/ml) (n = 5 in each group). Data represent mean ± SEM. After 48 h, vaginal muscularis was harvested and analyzed for levels of endogenous or recombinant FBLN5. (**A**) Upper blot was incubated with anti-FBLN5 whereas the lower blot was incubated with anti-His tag antibody to recognize recombinant FBLN5. (**B**) Quantitative analysis of endogenous FBLN5 in non-injured and injured vagina treated with HG or HG + FBLN5. *P < 0.05 compared with control (**C**) Immunoblot analysis of urea extracts of muscularis from control uninjured rats before and 12, 24, 48 h and 7D after cut injury. The 26 kDa bands represent nonspecific binding to IgG. (**D**) Quantitative analysis of FBLN5 levels in control uninjured (Ctl) or injured animals as a function of time after cut (open symbols) or wedge injury (solid squares). Data represent mean ± SEM. Uninjured control rats (n = 6) are presented with 20 cut and 12 wedge injuries divided equally among the four time points At 7 days after injury, horizontal line represents mean, individual values are presented. Data represent mean ± SEM, ^*^p < 0.05 compared with control; ^**^p < 0.05 compared with wedge.

Previous work indicated that hydrogel (HG)-containing recombinant FBLN5 injected into the vaginal wall of noninjured *Fbln5* null mice results in increased FBLN5 content and decreases in vaginal MMP-9^21^. To determine if HG-containing FBLN5 could rescue injury-induced upregulation of MMP-9, control cycling rats underwent surgical injury of the posterior vaginal wall and injected with either HG alone or 1 µg of rFBLN5-containing HG (100 µl of 10 µg/ml, Fig. 1). Surgical injury resulted in significant loss of glycosylated endogenous vaginal FBLN5 48 h after injury (65 kDa, Fig. 1A). Further, exogenous rFBLN5 (50 kDa) was also lost with non-detectable levels after injury (Fig. 1A,B). These findings led us to investigate the role of injury in regulation of vaginal FBLN5. A time course was conducted with two models of surgical injury as described in Methods. In intact cycling controls, FBLN5 was readily detected in the urea-extracted matrix of the distal posterior vaginal wall (Fig. 1C). Twenty-four h after injury, vaginal FBLN5 decreased dramatically from baseline (Fig. 1C,D) with limited recovery by 48 h. Recovery occurred to a variable extent 7 d after injury. Interestingly, both incisional (cut) and excisional (wedge) wounds resulted in similar injury-induced patterns of FBLN5 (Fig. 1D).

### Effect of systemic or local estrogen on baseline FBLN5 in the vaginal wall

**Figure 2:**
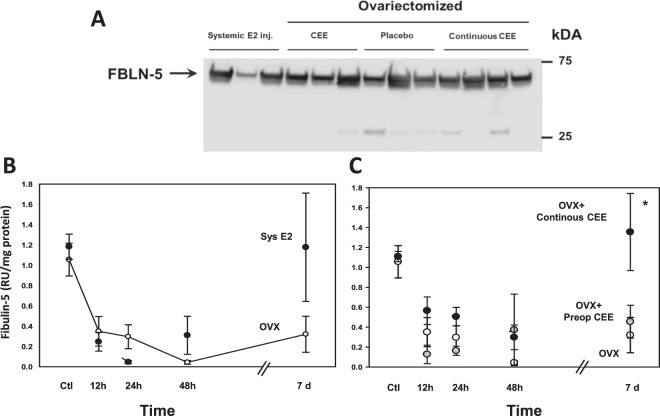
Effect of systemic or local estrogen on vaginal FBLN5. (**A**) Representative immunoblot of baseline levels of FBLN5 in uninjured control rats treated with systemic estradiol injections (Systemic E2 inj., 50 µg/kg/d), or ovariectomized with either (i) vaginal conjugated equine estrogen (CEE) cream discontinued prior to vaginal wall injury, (ii) placebo cream, or (iii) continuous vaginal conjugated equine estrogen (CEE) cream continued following vaginal wall injury. (**B**) Effect of systemic E2 on FBLN5 levels after injury. Data represent mean ± SEM of 5 animals at each time point in both groups. (**C**) Effect of local CEE cream on FBLN5 levels after injury. Ovariectomized animals treated with placebo (OVX), 2 weeks of preoperative CEE cream (OVX + Preop CEE) or preoperative CEE cream + continuous cream after injury (OVX + Continuous CEE). Results represent mean ± SEM of 5 animals at each time point in each group. *P < 0.05, ANOVA.

Next, we sought to determine if estrogen modified surgical-induced loss of vaginal FBLN5. In uninjured control rats, local or systemic estrogen did not affect basal levels of FBLN5 (Fig. 2A). To determine the impact of estrogen on FBLN5 after injury, ovariectomized rats were treated with either systemic or local conjugated equine estrogens as a function of time after injury (cut). Recovery of vaginal FBLN5 7 d after injury was not as robust in ovariectomized animals compared with intact cycling animals (Fig. 2B vs Fig. 1D). Treatment with systemic estrogen did not alter the decline in vaginal FBLN5 in the short-term, but increased FBLN5 levels in the late phase of wound healing (7d, Fig. 2B). Likewise, local treatment with vaginal conjugated equine estrogen (CEE) cream before and after simple vaginal wall injury did not alter acute downregulation of FBLN5 after injury (Fig. 2C). Further, preoperative vaginal CEE did not improve the poor recovery of vaginal FBLN5 in ovariectomized animals. Interestingly, long term recovery of vaginal FBLN5 levels after injury was improved not only with systemic estrogen but also with continuing CEE cream after injury (Fig. 2C).

### Effect of protease inhibitors on FBLN5 content in the vaginal wall

**Figure 3:**
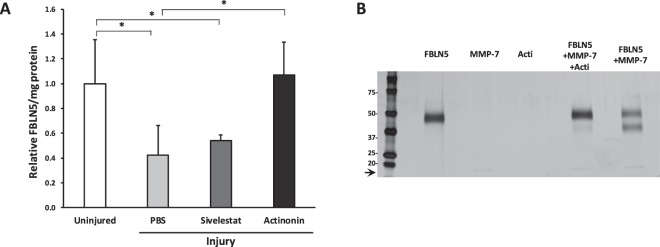
Effect of two protease inhibitors (sivelestat and actinonin) on FBLN5 content in the rat vaginal wall 24 hours after injury. (**A**) Animals were injected with PBS, sivelestat, or actinonin, at the time of injury. Data represent mean ± SEM levels of FBLN-5 of 4 animals per group. *One-way analysis of variance (P < 0.05). (**B**) Silver-stained gel of rFBLN5 (200 ng), MMP-7 (1:36), actinonin (acti), FBLN5 + MMP-7 ± acti, or FBLN5 + MMP-7 incubated at 37 °C for 4 min. 17 kDa protein represents MMP-7 (arrow).

The failure of estrogen to regulate the acute phase of injury-induced loss of FBLN5 led us to seek an alternate approach in which protease inhibitors could be injected directly at the time of injury. Based on experiments demonstrating that both MMP-7 and serine elastase cleaved FBLN5 *in vitro*^26^, we tested whether a broad-spectrum MMP inhibitor (actinonin) or the serine protease inhibitor sivelestat could prevent injury-induced loss of FBLN5 in the vaginal wall. Virgin cycling rats were injected with PBS, sivelestat or actinonin directly on both sides of the vaginal wall incision at the time of surgery. Twenty-four hours after injury, animals were sacrificed and matrix protein extracted with urea. Actinonin, but not sivelestat, blocked injury-mediated degradation of FBLN5 (Fig. 3A). Since MMP-7, but not MMP-9, degraded fibulin-5 *in vitro*^26^, we tested whether actinonin was effective in ameliorating MMP-7-mediated proteolysis of fibulin-5 (Fig. 3B). In agreement with previous studies, MMP-7 converted 50 kDa rFBLN5 into a 37 kDa fragment (Fig. 3B). Actinonin completely blocked MMP-7-induced degradation of FBLN5 (Fig. 3B).

**Figure 4:**
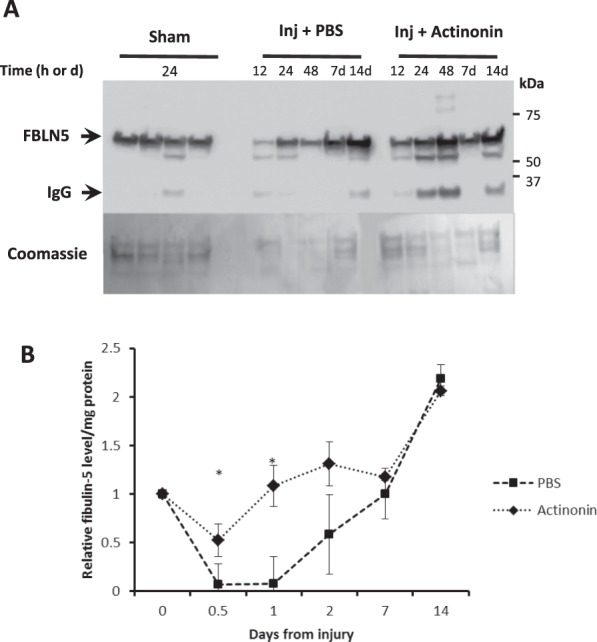
Effect of actinonin on FBLN5 content in the injured vaginal wall as a function of time. (**A**) Representative immunoblot and coomasie stained protein gel. 30 µg urea-extracted protein per lane. IgG represents nonspecific interacting protein without primary antibody. (**B**) Data represent mean ± SEM of 8 animals in each group. *Two-way analysis of variance (P < 0.05).

Next, we investigated the effect of actinonin on FBLN5 content as a function of time after injury in intact cycling rats (12, 24, 48 hours, and 7 and 14 days after injury). As expected, injury resulted in transient decreases in FBLN5 content until 7 d (Fig. 4A,B). Vaginal FBLN5 levels were maintained in animals injected with actinonin (Fig. 4A,B). Interestingly, in both groups, FBLN5 levels recovered to suprabasal levels 14 d after injury.

### Actinonin alters biomechanical properties of the vaginal wall

**Figure 5:**
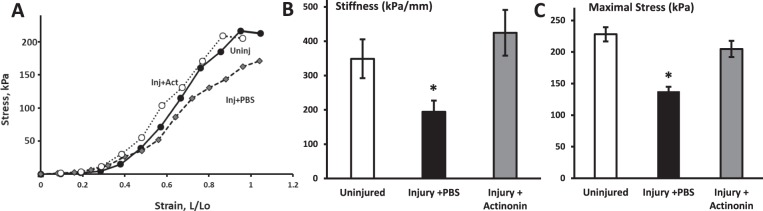
Effect of actinonin (Act) on biomechanical properties of the injured vaginal wall 7 days after injury. (**A**) Sample stress-strain relationships for vaginal tissue from uninjured controls (solid circles), injury + PBS (grey diamonds), and injury + actinonin (open circles). Quantification of maximal stress (**B**) and stiffness (kPa/mm, **C**). Results represent mean ± SEM of 8 animals in each group, except 1 control in which maximal stress was not reached. *P < 0.05, ANOVA.

To determine whether actinonin altered biomechanical properties of the injured vaginal wall, stress-strain relationships were conducted on rings of vaginal tissue obtained at the distal vagina from uninjured animals and injured animals treated with PBS or actinonin 7 days post injury (Fig. 5). Maximal stress (an index of tissue strength) was decreased significantly 7 d after injury with PBS. Actinonin rescued this loss of tissue strength. Likewise, stiffness was decreased 7 d after injury in the PBS group relative to uninjured sham animals, and actinonin rescued this loss of stiffness (Fig. 5B). Maximal strain, maximal vaginal length, and resting length were similar among treatment groups (Table 1).

**Table 1:** Biomechanical properties of the vaginal wall 7 d after injury with or without actinonin.

	Sham n = 7	Inj + PBS n = 8	Inj + Actinonin n = 8	p-value
Maximal distention, mm	12.4 ± 1.13	11.2 ± 0.85	9.6 ± 0.68	0.051
Resting length, mm	9.5 ± 1.2	9.3 ± 0.30	9.12 ± 0.38	0.941
Maximal strain, Fx increase in resting length	1.39 ± 0.15	1.21 ± 0.10	1.00 ± 0.07	0.069

### Histomorphology of the vaginal wall after injury

**Figure 6:**
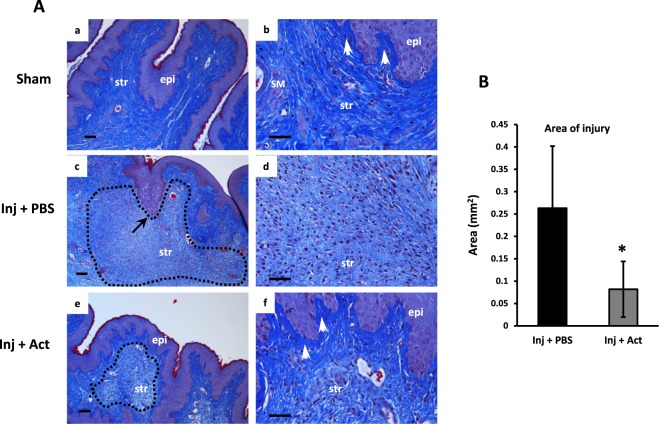
Effects of actinonin (Act) on histomorphology of the injured vaginal wall. (**A**) Vaginal wall from uninjured cycling rats (Sham, low and high magnification, a,b) or injured rats injected with PBS (mid panel, c,d) or Act (lower panel, e,f). Tissue was harvested 7 days after injury and stained with Masson’s trichrome stain. Images represent consistent results among 4 animals in each group. Dotted line represents area of injury. Arrow in panel c represents absence of lamina propria. Epi, epithelium; str, stroma; SM, smooth muscle, white arrows, subepithelial collagen. Bar = 100 µm. (**B**) Quantification of area of injury. Mean ± SEM, 4 animals per group < 0.05.

Masson’s trichrome staining was used to evaluate the impact of vaginal injury on collagen distribution and histomorphology (Fig. 6). In sham-operated animals, the vaginal wall was comprised of an epithelial layer, multiple rugae, and a dense underlying collagen network. Specifically, collagen staining (blue) in the underlying subepithelial space of the lamina propria was thickened and compact (Fig. 6a,b). Although looser than the lamina propria, collagen bundles remained dense and tightly organized in the muscularis (Fig. 6b) and inflammatory cells were rare. Consistent with previous reports^25^, a large area of injury persisted seven days after injury in PBS-treated animals (Fig. 6c) comprised of numerous inflammatory cells (predominantly macrophages by morphology) and loose collagen fibrils (Fig. 6d). Interestingly, the compact subepithelial collagen layer was absent at the interface between epithelium and stroma at the site of injury (Fig. 6d). Treatment with actinonin at the time of injury (Fig. 6e,f) resulted in (i) a smaller area of injury (Fig. 6B), (ii), grossly obvious decrease in the number of infiltrating immune cells, and (iii) restoration of the dense subepithelial collagen layer (Fig. 6f).

**Figure 7:**
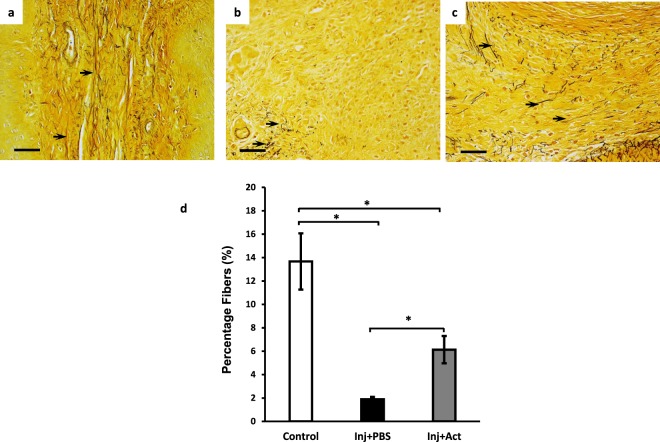
Effect of actinonin on elastic fibers after injury. Hart’s staining of vaginal stroma of rats before injury **(a)**, and 7 days after injury treated with PBS **(b)** or actinonin **(c)**. Black arrows denote elastic fibers. **(d)** Quantification of elastic fiber area in posterior vaginal wall of control animals, or 7 d after injury without (Inj + PBS/solid bar) or with actinonin (Inj + Act/gray bar) as described in Materials and Methods. Results represent mean ± SEM of 5 animals in each group. *P < 0.05, ANOVA.

Significant rescue of injury-induced loss of FBLN5 led us to examine elastic fibers after injury. Hart’s staining revealed that elastic fibers were numerous throughout the lamina propria and muscularis in uninjured sham animals. The fibers were long, branched, and extended from the subepithelial layer to the inner muscularis (Fig. 7a) covering 13.7% of the total cross sectional area. In PBS-treated animals, the absence of elastic fibers at the site of injury was striking. Small truncated fibers could be seen on high magnification at the periphery of the injured area (representing 1.9% of the cross sectional area) (Fig. 7b,d). Perhaps the most dramatic effect of actinonin was the appearance of numerous long elastic fibers at the site of injury together with truncated fibers suggesting either regeneration of elastic fibers or decreased degradation of fibers at the injury site (Fig. 7c,d).

## Discussion

The increased failure rate from reconstructive pelvic surgery for pelvic organ prolapse has resulted in an aggressive search for surgical adjuncts including numerous new materials of allografts, xenografts, and mesh^27^, many of which have proven to be associated with significant complications. We and others have used animal models to evaluate the effects of natural biologics on vaginal wound healing including pre- and post-operative estrogen^19,23–25^ and mesenchymal stem cells^25^. During these studies, inflammatory infiltrates in the vaginal wall after injury and slow time course of healing were remarkable. Although the epithelium quickly healed and appeared intact, the underlying muscularis and biomechanics of the vaginal wall remained compromised. These findings led us to consider the role of pre- and postoperative local estrogen which has been shown to increase protease inhibitors in the vaginal wall or if protease inhibitors directly could provide an adjunct to surgical wound healing. Although vaginal estrogen has been shown to regulate basal vaginal proteases and protease inhibitors^24^, estrogen did not regulate of the loss of vaginal FBLN5 in the acute phase of wound healing although systemic and continuous local application of CEE regulated the rate of recovery of FBLN5 in the late phase after injury. In contrast, broad-spectrum MMP inhibitor (actinonin) (i) prevented injury-induced transient downregulation of FBLN5 in the vaginal wall, (ii) rescued injury-induced loss of vaginal strength, and (iii) demonstrated positive site-specific effects on collagen distribution within the vagina.

### FBLN5: Decreased synthesis vs degradation

We concluded that FBLN5 was degraded in the vaginal wall after injury based on rapid loss of FBLN5 after injury (within 12 h) and rescue by the protease inhibitor actinonin. FBLN5 degradation has been described in other pathological conditions. As reported by Vierkotten, *et al*., degradation of FBLN5 results in increased risk of age-related macular degeneration, by way of fragmentation of Bruch’s membrane^28^. Hirai, *et al*., indicated that truncated FBLN5, and thattruncated FBLN5 does not promote elastogenesis, and this version of the protein increases with age as full-length FBLN5 decreases^29^. Previously, we found that the serine protease PRSS3 cleaved FBLN5 *in vitro*^8^. This protease, however, was not expressed in the rat vagina. Our work^26^, together with those Djokic *et al*.^30^, indicate that MMP-7 and MMP-12 (macrophage elastase) cleave FBLN5 *in vitro*. These proteases are highly expressed in immune cells with various insults^31–34^ and may mediate injury-induced loss of FBLN5. The inflammatory infiltrate, however, remained prominent 7 days after injury, a time in which FBLN5 is returning to normal levels. It is likely, therefore, that natural protease inhibitors may resolve some transient disruption of FBLN5 in the matrix. Actinonin seems to play a major role in prevention of early degradation. Although identification of the exact protease and its regulation are currently under investigation, results reported herein indicate that actinonin blocks MMP-7-induced degradation of FBLN5 *in vitro*. MMP-7-induced generation of the degradative product *in vitro* was not observed after vaginal injury most likely due to the presence of numerous other proteases *in vivo* capable of cleaving the degraded fragment into smaller peptides readily cleared within the wound.

### Actinonin, matrix dynamics, and biomechanical properties

Like skin and blood vessels, the vagina is subjected to continuous stresses acting in multiple directions. The mechanical properties of most connective tissues are dominated by the matrix in which fibrillary collagens and hydrated proteoglycans resist tensile and compressive forces whereas elastic fibers confer both compliance and passive recoil. Histomorphology indicated that actinonin was particularly effective in restoring or preventing disruption of the dense collagen network adjacent to the epithelial basement membrane. The increased maximal stress and stiffness with actinonin is likely due to this remarkable increase in localized collagen content beneath the basement membrane. Further, acute loss of elastic fibers at the site of injury was at least partially rescued by actinonin. Unlike the continuous synthesis and recycling of intracellular proteins, elastic fibers are extremely stable in uninjured resting adult tissues. The unique molecular structure, tissue distribution and longevity of elastic fibers predispose them to accumulate damage in aging dermal, pulmonary and vascular tissues^35^. The pro inflammatory effect of elastin degradative peptides may accelerate the inflammation process and impede wound healing^36^. Pelvic organ prolapse may join the list of aging elastic-fiber related diseases and dysregulation of the balance between synthesis and degradation. Local use of protease inhibitors to prevent limited loss of elastic fibers may increase longevity of pelvic organ support and improve risks of prolapse after vaginal delivery or reconstructive pelvic surgery.

### Summary and perspectives

In summary, we conclude that local injection of the broad-spectrum MMP inhibitor actinonin at the site of injury, but not estrogen, rescues acute loss of vaginal FBLN5 and improves histomorphology and biomechanical properties of the vagina in the short term. Injections were well-tolerated with no overt complications. It should be emphasized that matrix remodeling after injury represents a complex balance between synthesis and degradation. Excessive protease activity has been associated with detrimental effects on rotator cuff injuries^37^, diabetic ulcers^38^, and cancer progression^39–41^. Additional studies are needed to test the long-term effects of local protease inhibition on vaginal structure, function, and resilience against further injury. This initial work indicates that local protease inhibitors may protect the vagina early in the healing process, a vulnerable time in which patient activity is increasing after surgery.

## Methods

All animals were handled and euthanized in accordance with the standards of humane animal care described by the National Institutes of Health Guide for the Care and Use of Laboratory Animals, using protocols approved by the Institutional Animal Care and UseCommittee (IACUC) of the University of Texas Southwestern Medical Center at Dallas. A total of 250 virginal cycling female Sprague Dawley rats (Charles River Laboratories) at 12 weeks of age were housed in IACUC-approved facilities under a 12-L:12-D cycle at 22 °C (104 for estrogen studies and 146 for protease inhibitor experiments)^25^.

### Hydrogel and recombinant fibulin-5

The posterior vaginal wall of young (2–4 months of age) Sprague Dawley rats was injured by creating a posterior wall incision from the introitus to mid-vagina through the epithelium and stroma. Thereafter, the injury site was injected submucosally with HG alone or 1 µg of recombinant FBLN5-containing HG (100 µl of 10 µg/ml) as described previously^21^. Recombinant FBLN5 contained a His tag and could thereby be discerned by His tag antibodies. Further, recombinant FBLN5 is not glycosylated and was differentiated from endogenous FBLN5 by its increased mobility on SDS gels (50 kDa compared with 65 kDa). Animals were anesthetized by isoflurane and monitored every 15 min × 1 h, then daily for adverse reactions. Vaginal tissue was harvested 48 h after injury.

### Treatment groups

**Figure 8:**
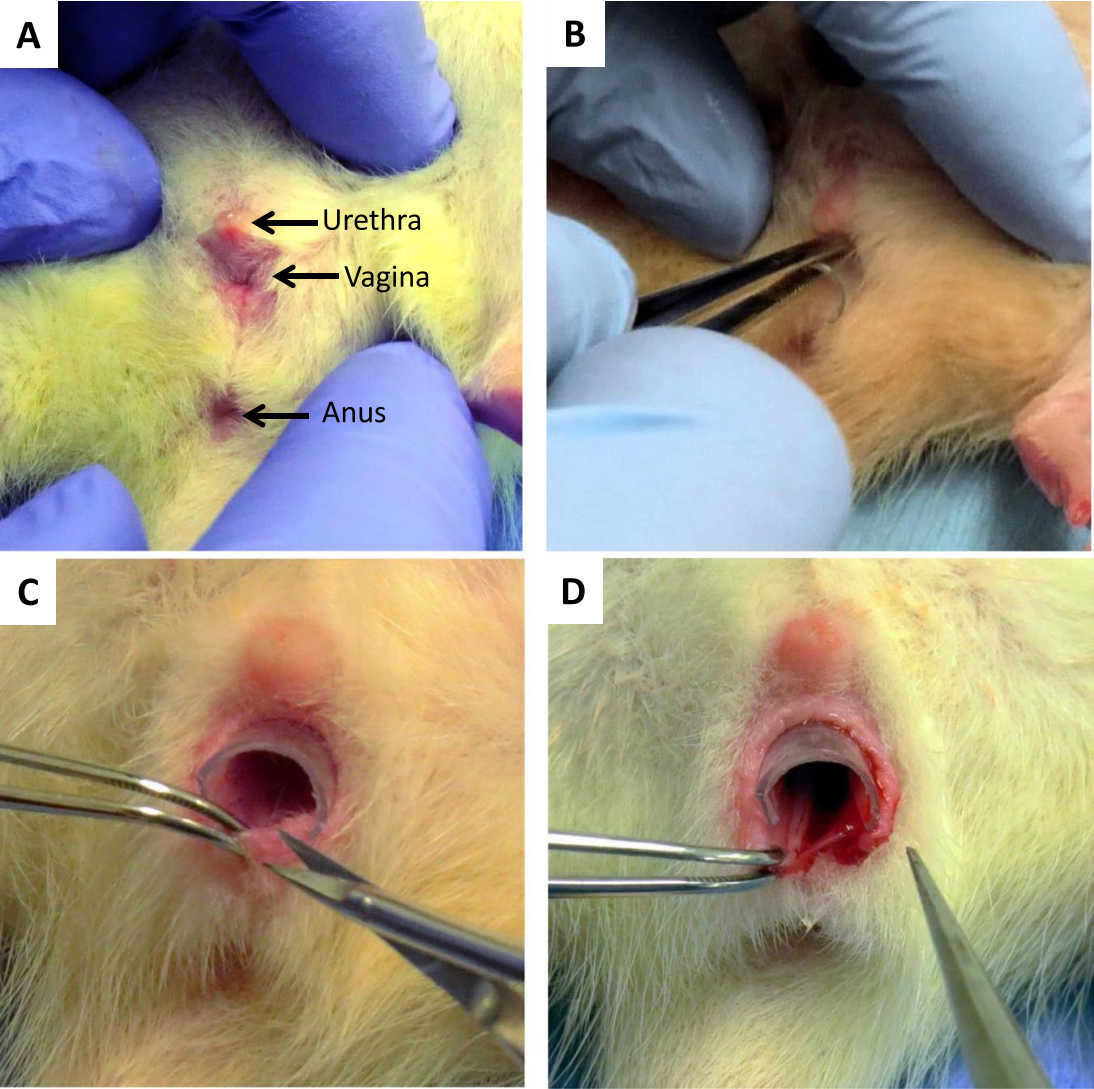
Posterior wall injury model. (**A**) Perineum of anesthesized rat showing urethra, vagina, and anus. (**B**) Insertion of vaginal speculum. (**C**) Posterior wall injury performed using microdissection instruments. (**D**) Posterior wall injury from distal to mid-vagina.

Vaginal injury was created by a posterior wall incision from the introitus to the mid vagina through the epithelium and stroma (Fig. 8)^25^. To optimize the impact of vaginal injury on FBLN5 levels, initial experiments were conducted with two types of vaginal injury using intact cycling rats. In another set of experiments, ovariectomized animals were used to control vaginal estrogen levels. Four conditions were used: systemic estrogen (50 µg/kg × 3 d, then every other day), placebo vaginal cream, preoperative low dose conjugated equine estrogen cream (CEE, 0.625 µg/d), and continuous CEE cream pre- and postoperatively (Supplemental Fig. 1). In each group, uninjured controls were compared with vaginal injury as a function of time. Uninjured control animals underwent isoflurane anesthesia alone with no vaginal wall injury. Cut injury consisted of a single linear incision in the posterior vaginal wall. The wedge injury involved excision of a triangular piece of the posterior vagina. Vaginal cream applications were administered using a tuberculin syringe. For protease inhibitor experiments, at the time of injury 40 µg of sivelestat (200 µl of 0.2 mg/mL), 100 µg actinonin (200 µl of 0.5 mg/mL) or phosphate buffered saline (PBS) was injected along the posterior wall injury using a 25 ga needle (100 µl on each side of the incision). Treatment groups were (1) sham (anesthesia, speculum placement, with no injury and euthanized at 24 h), (2) injury + PBS, (3) injury + sivelestat, and (4) injury + actinonin.

### Tissue processing

After euthanasia, the abdominal cavity was opened, the pubic symphysis disarticulated, and the uterine horns, bladder, cervix, and vagina dissected down to the perineal skin. Using microsurgical instruments and a dissection microscope, the perineal skin was removed and the bladder and urethra separated from the anterior vaginal wall. Uterine horns and cervix were removed from the vagina^19,25,42^. For animals randomized for histological analysis and biomechanical testing two rings were collected from the distal vagina. The vagina of animals randomized to immunoblotting or hydroxyproline quantification was opened longitudinally (Supplementary Figure), the epithelium was scraped off with a scalpel, and the posterior one-third collected, snap frozen in liquid nitrogen and stored at −80 °C. Specifically, the injured muscularis was comprised of the site of injury within 5 mm on either side.

### Matrix protein extraction

Frozen vaginal tissue was pulverized with a liquid nitrogen-chilled mortar and pestle. Tissue powder was then homogenized in basic buffer containing protease inhibitors (PIs) (16 mM potassium phosphate, pH 7.8, 0.12 M NaCl, 1 mM ethylenediaminetetraacetic acid, 0.1 mM phenylmethylsulfonyl fluoride, 10 μg/ml pepstatin A, and 10 μg/ml leupeptin) and centrifuged at 10 000 × *g*. The supernatant was then removed, and the previous homogenization step repeated after resuspending the remaining tissue pellet in basic buffer. After removal of the second supernatant, the remaining tissue pellet was suspended in 6.0 M urea in the above basic buffer, homogenized, and placed on a rotating rack for overnight extraction at 4 °C. The samples were then centrifuged (10 000 × *g* for 30 min), and the supernatant removed. Protein concentrations were determined using a bicinchoninic acid protein assay with standard curves of bovine serum albumin in appropriate buffers^19,25,42^.

### Immunoblot analysis

Urea-extracted protein samples (15 μg/lane) were applied to 4–20% gradient polyacrylamide gels (Bio-Rad), separated by electrophoresis, and transferred to nitrocellulose membranes. Identical gels were run side-by-side and Commassie blue-stained for protein loading comparison among the samples. After protein transfer, membranes were treated with blocking buffer, tris (hydroxymethyl) aminomethane 0.01 M, NaCl 0.15 M, Tween 20, 0.1%, pH 7.4 (TBS-T) with 2.5% nonfat milk for 1 h. Membranes were incubated in primary antibody, rabbit anti-rat FBLN5 (BSYN1923) at 1:250 dilution, overnight at 4 °C and then serially washed with TBS-T, followed by treatment with secondary antibody (goat immunoglobulin G anti-rabbit, 1:8000) at room temperature for 1 h. Membranes were serially washed with TBS-T and subsequently incubated with Western Lightning Chemiluminescence Reagent Plus (Perkin-Elmer) for 2 min. Signal strength was captured using a Fujifilm FLA 5100 image capture system. Protein band density was calculated using Image J software (ImageJ 1.46r, NIH) and normalized to total protein loaded quantified on Coomassie-stained gels^19,25,42^.

### Biomechanical testing

Biomechanical properties of individual vaginal rings were quantified as previously described^19,43^. Each vaginal ring was suspended between two stainless steel wire mounts and attached to a steel rod apparatus with a calibrated mechanical drive and to a force transducer. Tissues were maintained in a physiologic salt solution in water baths at 37 °C with 95% O_2_ and 5% CO_2_. After acclimation for 15 min, each ring was equilibrated to slack length (ring length at resting tone) as measured by the calibrated mechanical drive. Rings were distended in 1 mm increments with 30-sec intervals between each increment to allow stabilization of forces before each subsequent distention. This process was continued until failure (ring breakage) or until plateau of force generation. Wet weights of vaginal rings were determined after testing. Stress (kPa) was calculated as maximum force per unit area and plotted against strain (change in length divided by slack length), producing a sigmoid-shaped curve. Elasticity, an index of tissue stiffness, was calculated from the slope of the linear portion of the curve. For distensibiliy, stress was plotted against deformation in mm. Distensibility was computed from the slope of the linear portion of the curve^19,25,42–44^.

### Histomorphology

Vaginal rings were fixed in neutral buffer formalin (10%). Tissues were subsequently processed and embedded in paraffin blocks. Cross-sections of each vaginal ring were stained with hematoxylin and eosin, Masson trichrome stain, and Hart’s stain^25^. Images of each section were captured and analyzed using a Nikon E1600 microscope and Nikon NIS Elements AR software (Melville, NY). Morphology was reproducible among 4 animals in each treatment group. ImageJ 1.52 software (NIH, Bethesda, MD) using a default threshold of “MaxEntropy” was used to analyze elastin tissue composition in the vaginal muscularis of Hart’s stained sections.

### Proteolytic cleavage assay

Recombinant human FBLN5 (R&D Systems #3095-FB, Minneapolis, MN, USA) was incubated with MMP-7 ± actinonin. All reactions were performed in a basic buffer (50 mM Tris-HCl, pH 7.4, 150 mM NaCl, 5 mM CaCl2, 0.1% TX-100, 0.01% brij L23) for 4 min. MMP-7 was added to rFBLN5 in a molar ratio of 1:36. Samples were boiled prior to loading on a 4 to 20% Criterion gradient polyacrylamide gels (Bio- Rad, Hercules, CA, USA). Thereafter the gel underwent silver staining^7,8^.

### Statistical analysis

Of the various endpoints in this study (biomechanics, fibulin-5 levels, histomorphology, and elastic fibers), biomechanical properties are the most variable. Thus, we powered the study based on biomechanics from data reported previously in injured rats^25^. To detect a 40% increase in vaginal stiffness with intervention with power of 0.8 and an α of 0.05, a sample size of 6 in each group would be required. We increased the number to 8 to account for comparisons between 3 groups, i.e., uninjured, injured + PBS, and injured + protease inhibitor. Analysis of variance (ANOVA) and nonparametric (Kruskal Wallis) testing was performed, as appropriate, for multiple group comparisons. Post testing between groups was performed using Holm-Sidak method for normally distributed data and Dunn tests for nonparametric distributions with uninjured/untreated animals as the control. P < 0.05 determined significance.

## Supplementary information

Supplementary material
