# Supplementary material for: Effect of Protease Inhibitors in Healing of the Vaginal Wall
Source: Sci Rep. 2019 Aug 26;9:12354. doi: 10.1038/s41598-019-48527-0 (PMC6710245; doi:10.1038/s41598-019-48527-0)

EFFECT OF PROTEASE INHIBITORS IN HEALING OF THE VAGINAL WALL

Maria Florian-Rodriguez[1] *MD, Kathleen Chin[1], MD, Jennifer Hamner[1], DO, Jesus Acevedo[1], Patrick Keller[1], and Ann Word[1] MD

[1]Cecil H and Ida Green Center for Reproductive Biological Sciences
Department of Obstetrics and Gynecology
Division of Female Pelvic Medicine and Reconstructive Surgery
University of Texas Southwestern Medical Center, Dallas, Texas, USA

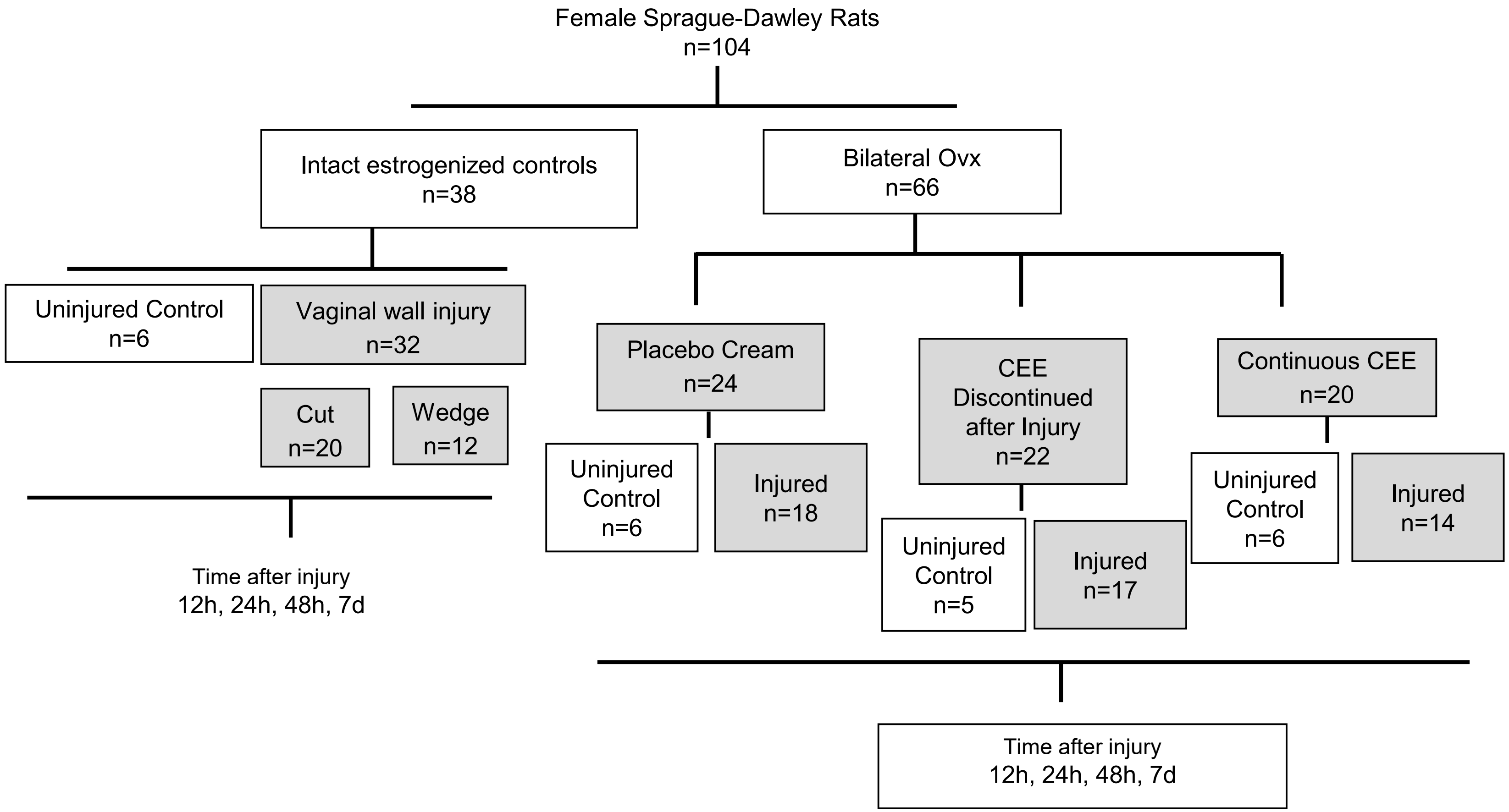

Florian-Rodriguez,
Supplementary Figure 1

Supplementary Figure 1. Flowchart of female Sprague-Dawley rat treatment groups. Systemic estradiol injections were administered 1 and 3 days prior to injury, the day of injury or anesthesia alone (uninjured control group), and every other day following vaginal wall injury. Cut injury consisted of a single linear incision in the posterior vaginal wall. Wedge injury involved excision of a triangular piece of the posterior vagina. In ovariectomized rats, daily cream was applied for two weeks beginning on the day of oophorectomy, and continued or discontinued following vaginal cut injury based on treatment assignment. Tissues were collected at four time points following vaginal wall injury from 12 h to 7 d.

Fig. 1A

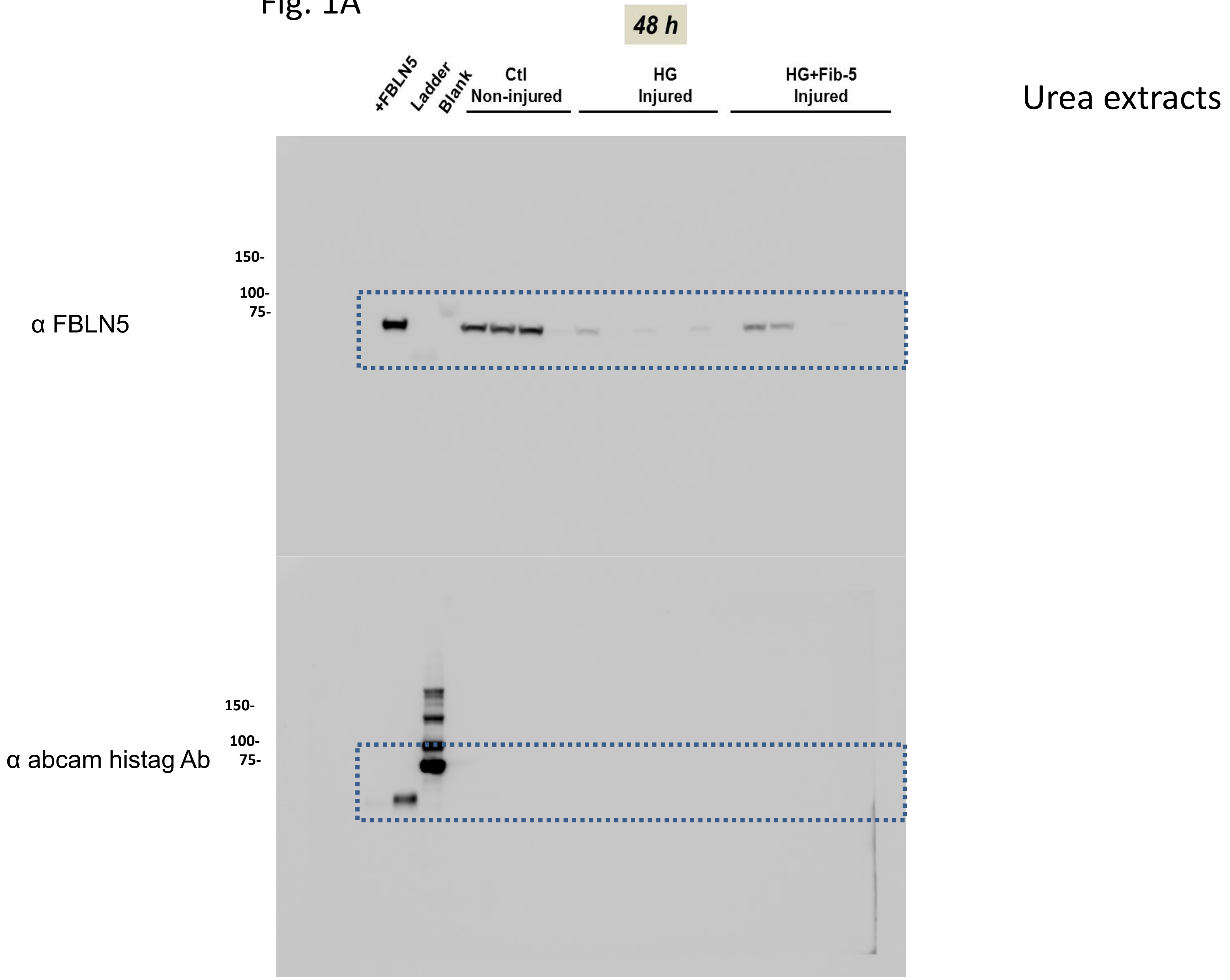
48 h
+FBLN5
Ladder
Blank
Ctl
Non-injured
HG
Injured
HG+Fib-5
Injured
Urea extracts
α FBLN5
150-
100-
75-
α abcam histag Ab
150-
100-
75-

Fig. 1C. Blot A

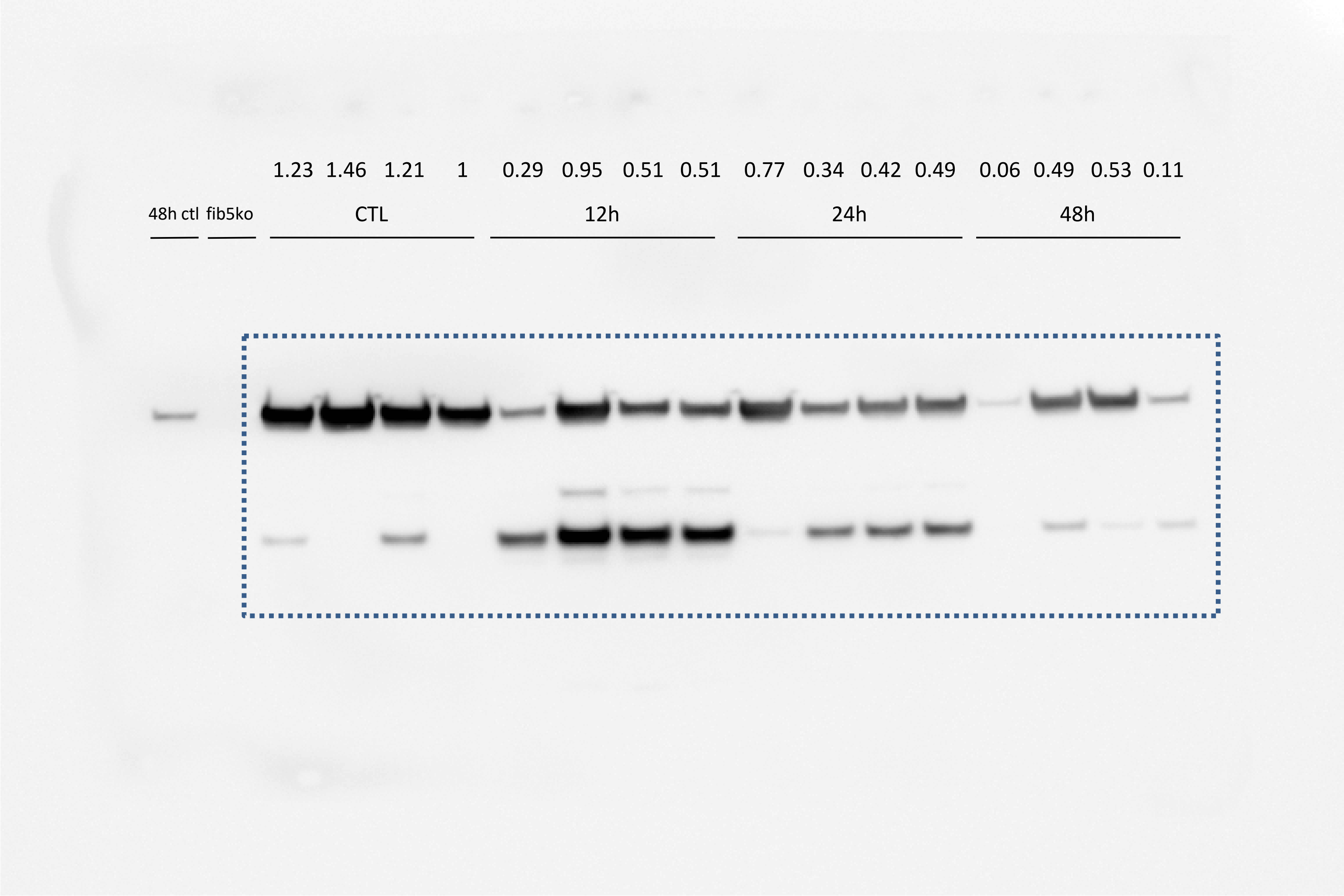
1.23 1.46 1.21 1 0.29 0.95 0.51 0.51 0.77 0.34 0.42 0.49 0.06 0.49 0.53 0.11
48h ctl
fib5ko
CTL
12h
24h
48h

Fig. 1C Blot A

Amido Black

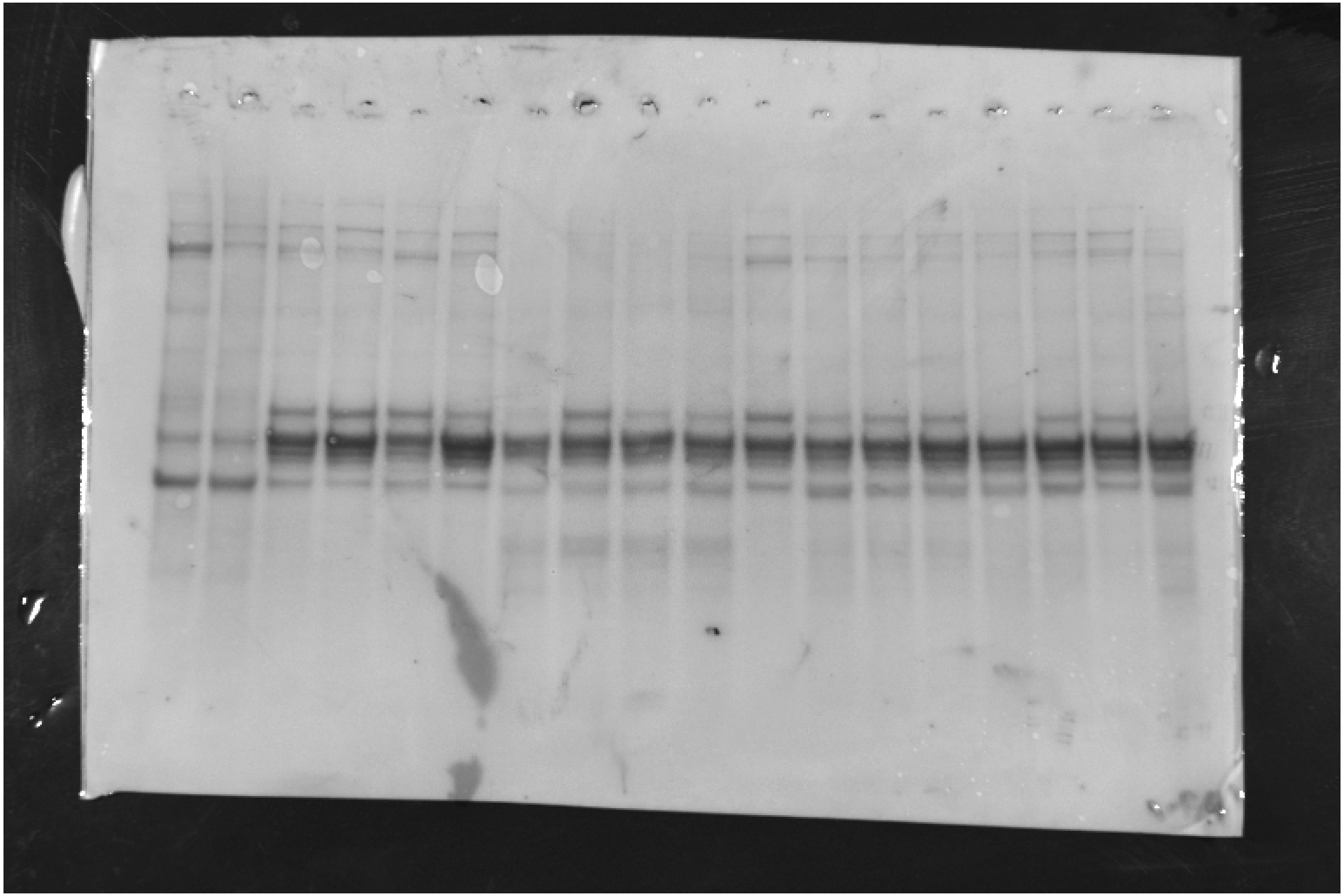

Fig. 1C blot B for 7d time point

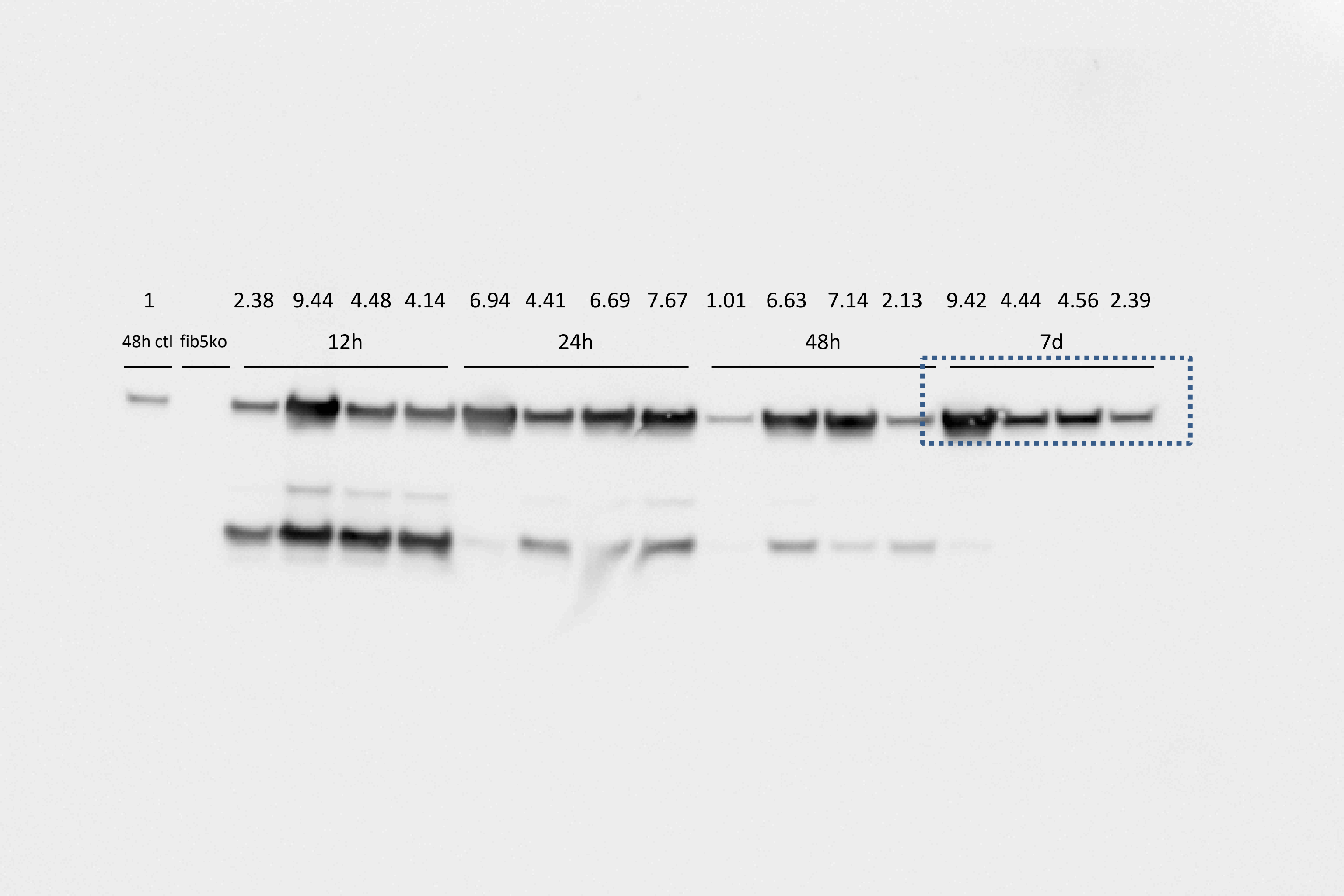

Amido Black Fig. 1C blot B for 7d time point

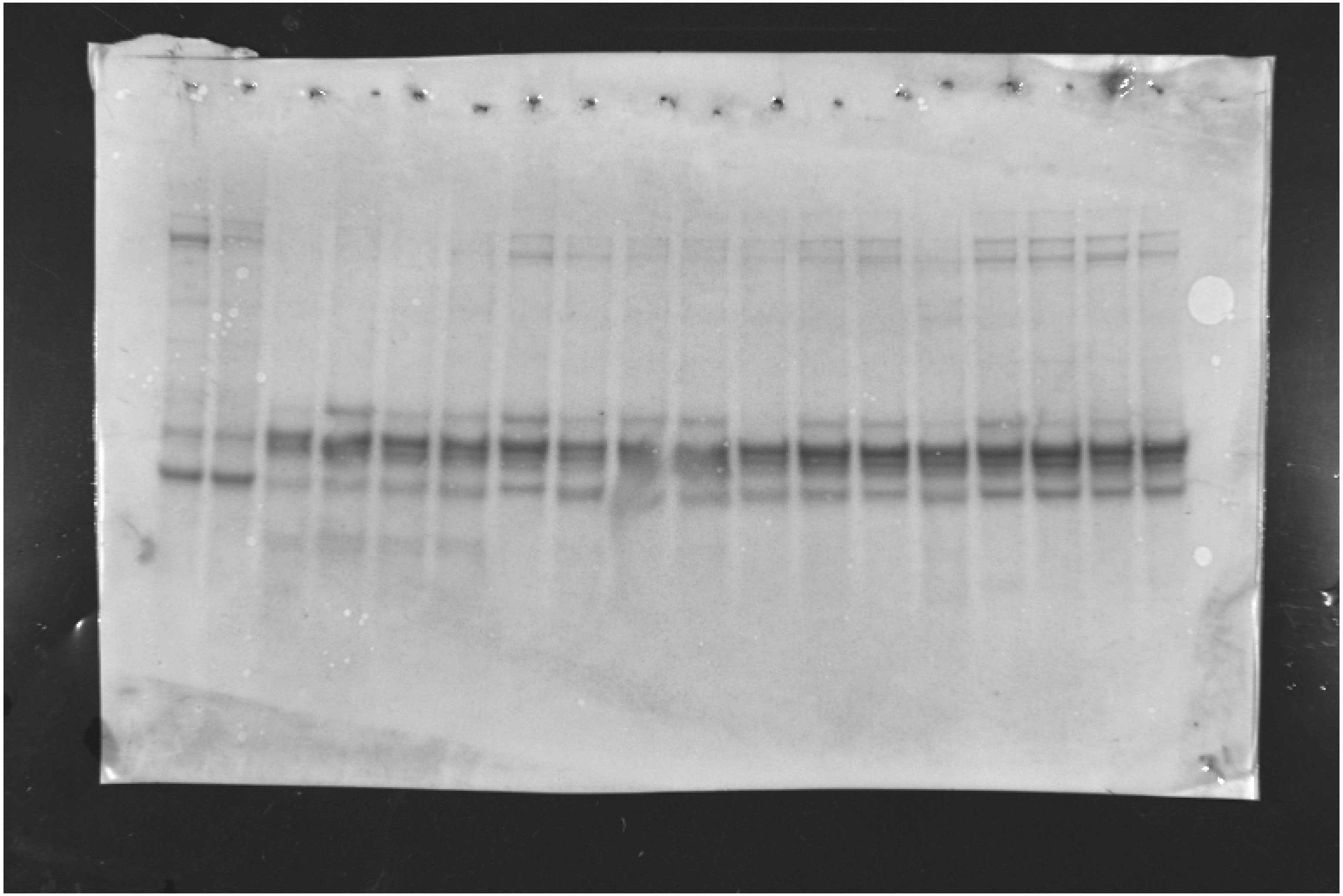

Fig. 2A

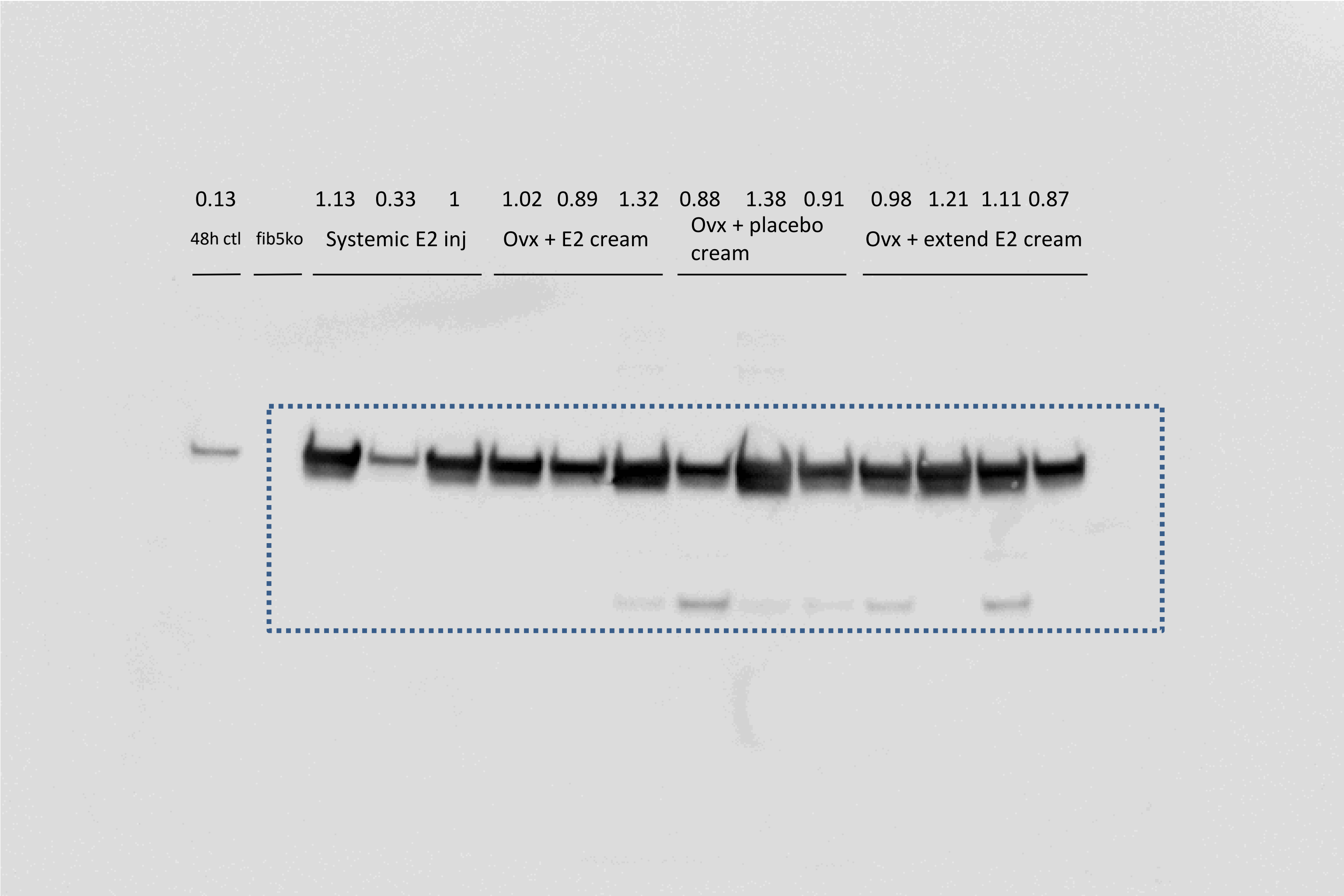
0.13
48h ctl
fib5ko
1.13 0.33 1
Systemic E2 inj
1.02 0.89 1.32
Ovx + E2 cream
0.88 1.38 0.91
Ovx + placebo cream
0.98 1.21 1.11 0.87
Ovx + extend E2 cream

Fig. 2A
Amido Black

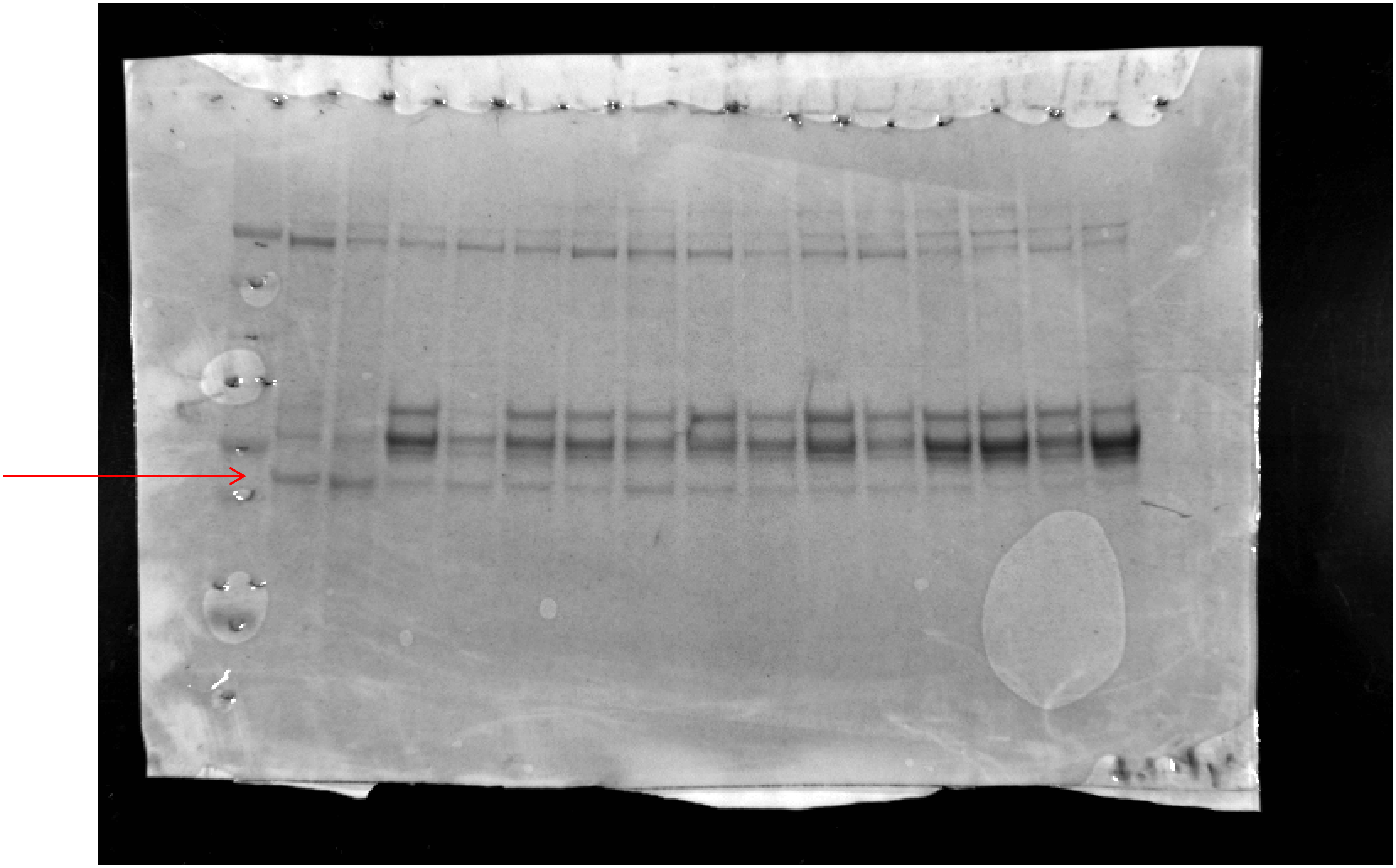

Fig. 3B

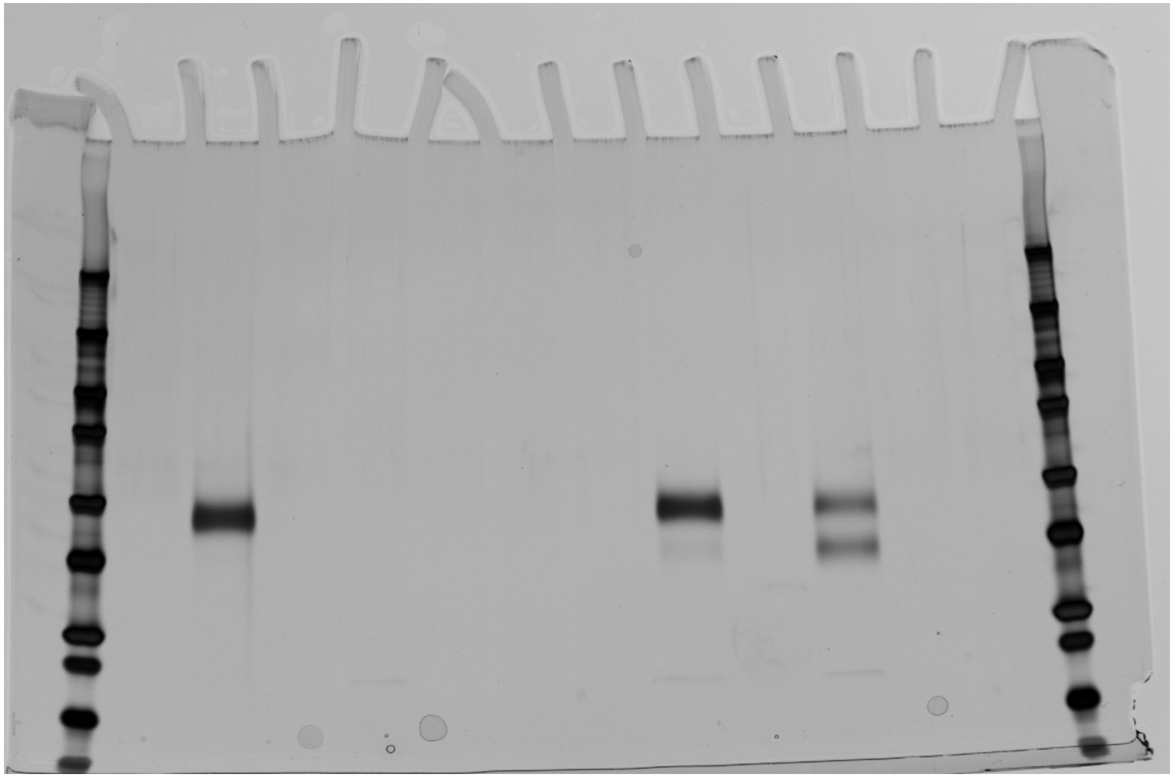

Fig. 4A

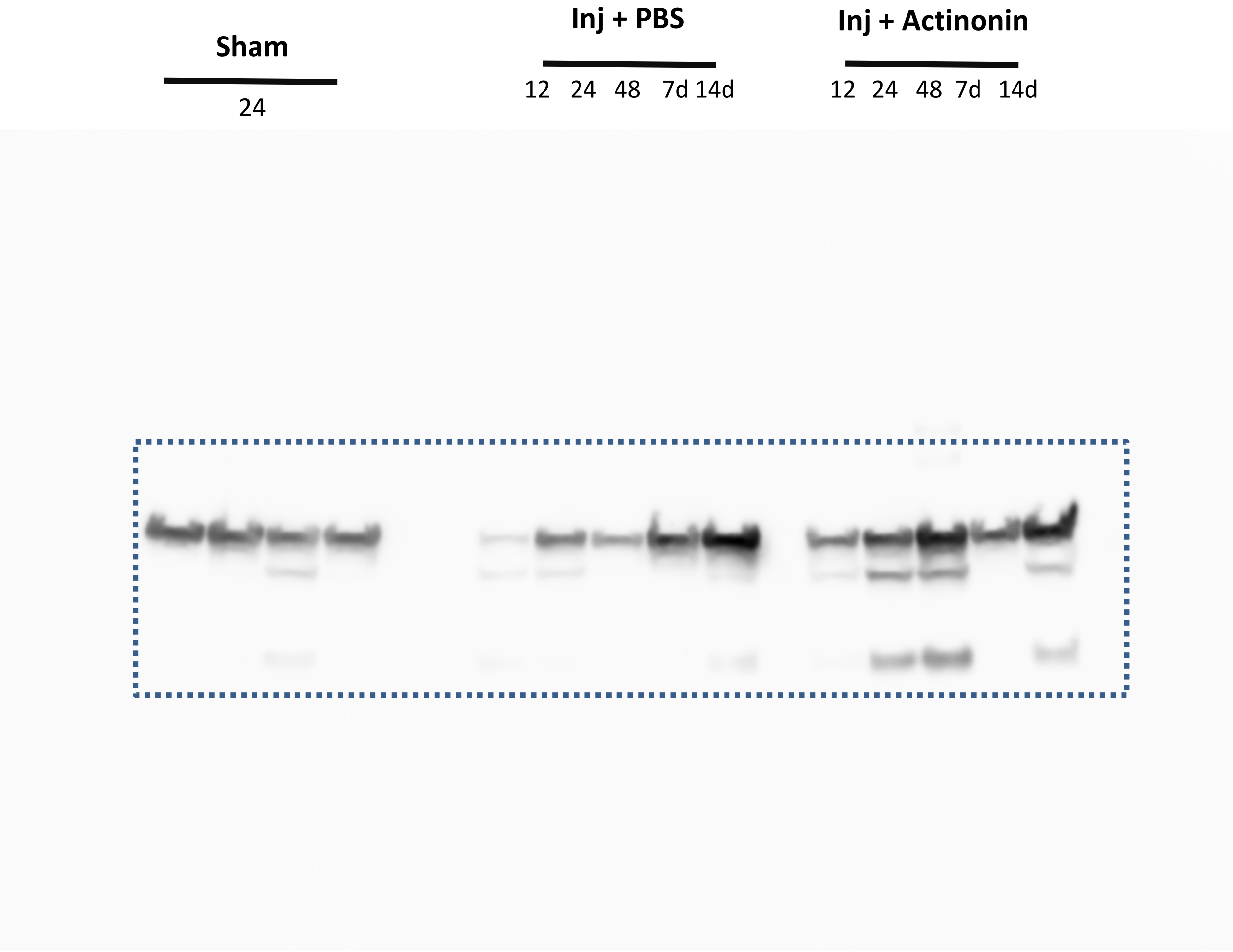

Fig. 4A
Amido Black

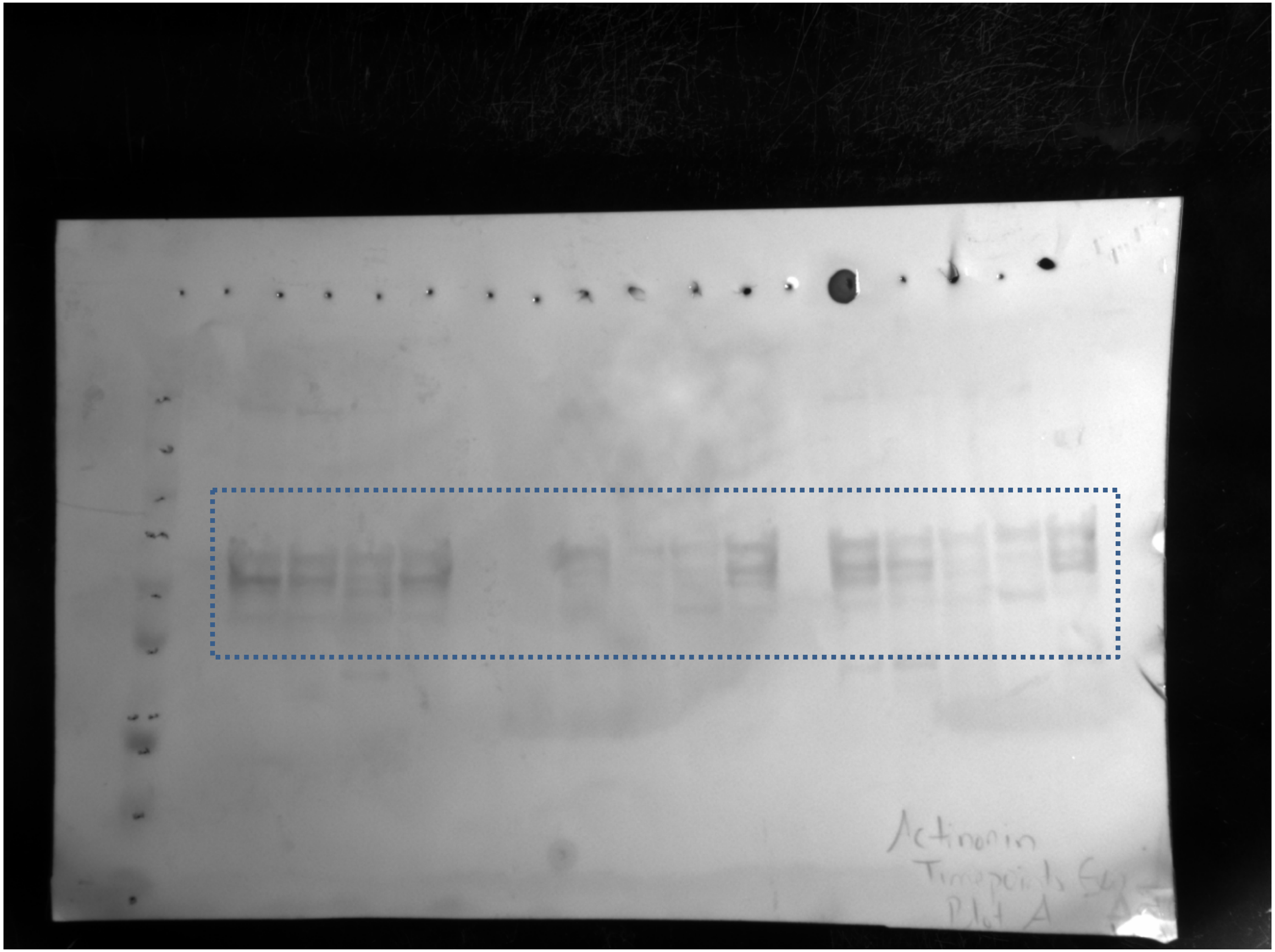

Supplement: Supplementary file 1 — Supplementary material [file 41598_2019_48527_MOESM1_ESM.pdf]
